# Xanthine dehydrogenase rewires metabolism and the survival of nutrient deprived lung adenocarcinoma cells by facilitating UPR and autophagic degradation

**DOI:** 10.7150/ijbs.78948

**Published:** 2023-01-01

**Authors:** Man-man Chen, Wei Guo, Si-meng Chen, Xiao-zhen Guo, Lan Xu, Xiao-yu Ma, Yu-xiang Wang, Cen Xie, Ling-hua Meng

**Affiliations:** 1 Division of Anti-tumor Pharmacology, Shanghai Institute of Materia Medica, Chinese Academy of Sciences, 501 Haike Road, Shanghai 201203, China.; 2 State Key Laboratory of Drug Research, Shanghai Institute of Materia Medica, Chinese Academy of Sciences, 501 Haike Road, Shanghai 201203, China.; 3 University of Chinese Academy of Sciences, Beijing 100049, China.

**Keywords:** Xanthine dehydrogenase, nucleoside degradation, cell survival, UPR, autophagy, LUAD

## Abstract

Xanthine dehydrogenase (XDH) is the rate-limiting enzyme in purine catabolism by converting hypoxanthine to xanthine and xanthine to uric acid. The altered expression and activity of XDH are associated with the development and prognosis of multiple types of cancer, while its role in lung adenocarcinoma (LUAD) remains unknown. Herein, we demonstrated that XDH was highly expressed in LUAD and was significantly correlated with poor prognosis. Though inhibition of XDH displayed moderate effect on the viability of LUAD cells cultured in the complete medium, it significantly attenuated the survival of starved cells. Similar results were obtained in XDH-knockout cells. Nucleosides supplementation rescued the survival of starved LUAD cells upon XDH inhibition, while inhibition of purine nucleoside phosphorylase abrogated the process, indicating that nucleoside degradation is required for the XDH-mediated survival of LUAD cells. Accordingly, metabolic flux revealed that ribose derived from nucleoside fueled key carbon metabolic pathways to sustain the survival of starved LUAD cells. Mechanistically, down-regulation of XDH suppressed unfolded protein response (UPR) and autophagic flux in starved LUAD cells. Inhibition of XDH decreased the level of amino acids produced by autophagic degradation, which was accompanied with down-regulation of mTORC1 signaling. Supplementation of amino acids including glutamine or glutamate rescued the survival of starved LUAD cells upon knockout or inhibition of XDH. Finally, XDH inhibitors potentiated the anti-cancer activity of 2-deoxy-D-glucose that induced UPR and/or autophagy in vitro and in vivo. In summary, XDH plays a crucial role in the survival of starved LUAD cells and targeting XDH may improve the efficacy of drugs that induce UPR and autophagy in the therapy of LUAD.

## Introduction

Lung adenocarcinoma (LUAD) is the most common and aggressive subtype of non-small cell lung cancer (NSCLC), and is the leading cause of cancer-associated mortality worldwide 1**.** Large-scale genomic studies have identified that somatic mutations in TP53, KRAS, KEAP1, STK11, and EGFR are frequent in LUAD 2, which have also been validated as oncogenic drivers. Selective targeting the oncogenic drivers such as EGFR and BRAF, ALK and ROS1 has achieved remarkable success. Recently, immune checkpoint inhibitors against PD-1 or PD-L1 have been also approved for treatment of LUAD 3. Despite these advances, there are still unmet medical needs in LUAD patients, who are unable benefitted from the current targeted therapy. Moreover, therapeutic resistance commonly occurred despite the initial response 4. Therefore, it is of great importance to identify new therapeutic targets and discover drugs with new mode of action for the treatment of LUAD.

Metabolic reprogramming has been recognized as one of the hallmarks of human cancer. Metabolic pathways are reshaped in tumor cells to enhance the acquisition and utilization of nutrients, sustain growth and proliferation 5. Purines not only act as the basic components of nucleotides that are building blocks for DNA and RNA, but also provide the necessary energy and cofactors for cell metabolism 6. Emerging evidences have implicated the deregulation of purine metabolism in cancer, and enhanced purine biosynthesis is associated with the progression of multiple types of cancer, including hepatocellular carcinoma (HCC), cholangiocarcinoma, glioblastoma and lung cancer 7-11. Metabolomic profiling of tumor tissues from a cohort of 33 LUAD patients revealed not only increased levels of ribose-5-phosphate levels in tumor tissue, which indicated an accelerated synthesis of purine required by highly proliferative cancer cells, but also a significant accumulation of multiple byproducts derived from purine catabolism including adenosine, inosine, hypoxanthine and xanthine 12. Similarly, metabolomic study of 39 pairs of malignant and nonmalignant lung tissue revealed altered nucleotide metabolism in early stage LUAD 13. Specifically, increased level of xanthine and uric acid were found in LUAD tissues, indicating elevated activity xanthine dehydrogenase (XDH) in LUAD tissues. These studies collectively indicated the up-regulation of purine degradation pathway in LUAD. However, the roles of purine degradation in LUAD are unknown.

XDH, also known as xanthine oxidoreductase (XOR) or xanthine oxidase (XO), is the rate-limiting enzyme acting in the last two steps of purine degradation, converting hypoxanthine to xanthine and xanthine to uric acid. XDH is a homodimer with a molecular mass of approximately 150 kDa for each subunit, which comprises two iron-sulfur redox clusters (Fe2-S2, 20 kDa), one flavin adenine dinucleotide (FAD, 40 kDa), as well as one molybdopterin (Mo-Co, 85 kDa) 14. The expression of XDH widely varies across different human tissues and is highly expressed in liver, breast and intestine 15. XDH has been recognized as a therapeutic target of gout as well as conditions associated with hyperuricemia 16. Recently, XDH and its byproducts are also considered to be involved in pathological processes such as metabolic syndrome 17, hemolytic diseases 18, cardiovascular disorders 19, and cancer 20. Mounting evidences demonstrate that XDH expression and activity are altered in various types of tumor compared to their normal counterparts 21. The expression and activity of XDH are often reduced in tumors derived from tissues with high XDH expression because of the poor differentiation of malignant cells, such as HCC, breast cancer and colon cancer, and low XDH level has been linked to aggressive phenotypes and unfavorable clinical outcomes 22-24. In contrast, increased levels of XDH are often observed in cancers originating from tissues expressing low level of XDH including prostate cancer, brain tumor and lung cancer, due to elevated demand for purine metabolism and the activation of the inflammatory response 25. Elevated byproducts of XDH have been found in both human LUAD and mouse LUAD models 13, 26. Combined analysis of the methylome and transcriptome revealed that XDH was one of the potential therapeutic targets for NSCLC with low expression of PD-L1 and wild-type EGFR 27. These findings indicate the potential role of XDH in LUAD, while the mechanism remains elusive.

In this study, we demonstrated that XDH was highly expressed in LUAD and was significantly correlated with poor clinical outcome. Knockout or inhibition of XDH attenuated UPR and autophagy in starved LUAD cells, which resulted in nutrients scarcity due to the blockade of the degradation of nucleosides and proteins, and finally cell death. These findings indicate that targeting XDH could be a potential strategy to improve the efficacy of anti-LUAD drugs that induce UPR or autophagy.

## Materials and methods

### Compounds

Febuxostat, allopurinol, topiroxostat, adenosine, guanosine, xanthine, hypoxanthine, Inosine 5'-monophosphate disodium salt hyprate, uridine and D-ribose were purchased from Sigma-Aldrich (St. Louis, MO, USA). Forodesine was purchased from MedChemExpress (NJ, USA). 2-Deoxy-D-glucose was purchased from Selleckchem (Houston, TX, USA). Glutamine and inosine were purchased from Sinopharm Chemical Reagent (Shanghai, China). Glutamate, aspartate, asparagine, serine, glycine, alanine, arginine, lysine, valine, histidine, methionine, tyrosine and leucine were purchased from J&K Scientific (Shanghai, China).

### Cell lines and cell culture

Normal lung fibroblast IMR-90 cells, and the lung adenocarcinoma cell lines HCC78, H23, CALU-1, CALU-6, H1755, H1650, H460, H1355, H1299, H1975, H358, H647, HCC44, H441, H2030, H2228, H322, H1838, H1792, H596, PC9 and A549 were obtained from the American Type Culture Collection (Manassas, VA, USA). IMR-90 cells were maintained in MEM medium supplemented with 1% MEM non-essential amino acids and 1% sodium pyruvate. A549 cells were grown in F-12 basic medium and the rest of cells were cultured in RPMI 1640 medium. All culture media were supplemented with 10% FBS and 1% penicillin/streptomycin. Cells were maintained in humidified incubators at 37°C with 5% CO_2_. Cell lines were authenticated by analyzing short-tandem repeats by Genesky Biotechnologies Inc (Shanghai, China).

For starvation assays, cells were cultured in Hank's balanced salt solution (HBSS) without glucose (formulations of HBSS were provided in Supplemental Table S1), which was used to maintain PH and osmotic balance as well as provide cells with essential inorganic ions.

### Immunohistochemistry staining of XDH

Immunohistochemistry staining of XDH was performed by Shanghai ZuoCheng Bio Company (Shanghai, China, No. HLug-Ade180Sur-01). Slides were photographed with a LeicDM6 B microscope equipped with sCMOS camera (Leica, Wetzlar, Germany). The staining was evaluated manually and graded using a two-score system based on intensity score and proportion score described previously 28.

### Cell viability assay

Cell viability was evaluated by Sulforhodamine B (SRB, Sigma-Aldrich, St. Louis, MO, USA) assay as described previously 29. Cells seeded in 96-well plate were treated in triplicate with febuxostat or allopurinol at 37°C for 72 h. Optic density was measured at 560 nm with Spectra Max190 (Molecular Devices, Sunnyvale, CA, USA). The relative cell viability was calculated using the following formula: (OD_treated cells_ / OD_control cells_) × 100%.

### Western blotting

Cells were harvested and lysed with RIPA buffer (Beyotime, Shanghai, China). Cell lysates were subjected to standard Western blotting using antibodies against GRP78, XBP1s, ATF-4, CHOP, p62, phosphor-mTOR (Ser 2448), mTOR, phospho-p70 S6 Kinase (p70S6K, Thr389), p70S6K, phosphor-4EBP1(Thr 37/36), 4EBP1 (Cell Signaling Technology, Danvers, MA, USA), XDH (Abcam, Cambridge, UK), and GAPDH (Proteintech, Rosemount, IL, USA). The β-actin and LC3 antibody were purchased from Sigma-Aldrich (St. Louis, MO, USA).

### RT-qPCR analysis

Total RNA were extracted from cells using RNAeasy Mini Kit (QIAGEN, CA, USA, No. 74106). RNA was reverse transcribed into cDNA using HiScript®ⅡQ RT SuperMix for qPCR (Vazyme, Nanjing, China, No. R223-01) and then used for real-time quantitative PCR using SYBR Green (Bio-Rad, California, USA, No. 172-5124). The sequences of the used primers are listed in Supplemental Table S2.

### GFP-RFP-LC3 assay

Virus expressing GFP-RFP-LC3 was obtained from Obio Technology (Shanghai, China). H460 cells was infected with the virus for 48 h, and then selected with puromycin (5 μg/mL) for 72 h. H460 cells expressing GFP-RFP-LC3 were seeded on slides in 24-well plate. Cells were fixed with 4% of paraformaldehyde and stained with DAPI. The slides were photographed with an Olympus BX51 fluorescent microscope (Olympus, Tokyo, Japan).

### Clonogenic survival assay

Clonogenic survival assay was performed as described previously 30 with minor modifications. Briefly, cells cultured in complete medium (RPMI 1640 with 10% FBS) were seeded in 12-well plates. When cell density reached about 80% confluent, cells were washed with PBS twice and further incubated in HBSS or complete medium for 18 h. Then the medium was replaced with complete medium and cells were cultured for additional 24 h. Cells were fixed with methanol for 30 min and stained with crystal violet. Crystal violet was dissolved with 33% acetic acid and the OD value was measured at 570 nm with Spectra Max190 (Molecular Devices, Sunnyvale, CA, USA).

### Intracellular amino acid and ATP/ADP/AMP analysis

Cells were collected in PBS and cell number was counted. Intracellular amino acids and AMP/ADP/ATP were extracted with methanol/water/chloroform mixtures. The aqueous phase was subjected to evaporation to dryness under vacuum. The level of amino acids and AMP/ADP/ATP were measured using an I-Class UHPLC coupled to a Q-TOF mass spectrometer (Waters Corp, Milford, MA, USA). The aqueous extracts were reconstituted with 65% acetonitrile and separated using a BEH amide column (ACQUITY UPLC BEH Amide 2.1x50 mm, i.d. 1.7 um, Waters). Q-TOF mass data was covered in a range of m/z 50 - 1500, with the resolution set at 22, 000.

### Generation of XDH-Knockout H460 cell line

Plasmids to induce CRISPR/Cas9-mediated XDH gene editing were constructed according to the protocol published by Zhang Feng laboratory 31, 32. Briefly, annealed sgRNA oligonucleotide pairs (Supplemental Table S3) were ligated into BsmBI-digested lentiCRISPRv2 with T4 ligase. LentiCRISPRv2 expressing sgRNAs were transfected into HEK293T cells with lentiviral packaging vectors pMD2.G and psPAX2 using lipo2000 transfection reagent (Invitrogen, California, USA). H460 cells were infected with the filtered lentivirus with polybrene (8 µg/mL). After incubation for 48 h, cells were selected in the presence of puromycin (5 μg/mL) for 72 h. Cells were then plated at a low density for a period of 1-2 weeks. Clones were picked and expanded. Confirmation of loss of XDH was confirmed by Western blotting.

### Metabolic flux analysis by UPLC-QTOF‑MS

Cells were cultured in HBSS medium supplemented with 2 mM [^13^C_5_] of ribose-inosine (No. NUC-072, Omicron Biochemicals, South Bend, USA) for 18 h at 37 °C. Cells were then washed three times in cold PBS and stored at -80 °C. Cells were lysed in 80% methanol aqueous solution containing 100 ng/mL of phenylalanine-2,3,4,5,6-d5 and lysine-4,4,5,5-d4 as internal standard compound, followed by sonication and centrifugation. The supernatants were concentrated in a vacuum centrifugal concentrator for 1 h, then reconstituted with 80% methanol solution. Metabolite profiling was detected by UPLC-QTOF‑MS as described previously 33. A LC-30AD series Ultra Performance Liquid Chromatography system (Nexera®, Shimadzu, Japan) and a Waters ACQUITY UPLC BEH Amide column (100 × 2.1 mm,1.7 μm) were used for UPLC-QTOF-MS with a SciexTripleTOF® 5600 plus mass spectrometer (Sciex, Concord, ON, USA). Data for metabolomics acquisition was performed in both positive (ESI+) and negative (ESI-) modes. The mass range scanned was m/z 50-1000 in full data storage mode. The instrument was mass calibrated by automatic calibration infusing the Sciex Positive Calibration Solution (No. 4460131, Sciex, Foster City, CA, USA) for positive mode and Sciex Negative Calibration Solution (No.4460134, Sciex, Foster City, CA, USA) for negative mode after every six-sample injection. One pooled quality control sample and one blank vial were run after every 10 samples.

### Animal studies

Animal experiments were performed in accordance with the Institutional Ethical Guidelines on Animal Care and were approved by the Institute of Animal Care and Use Committee at Shanghai Institute of Materia Medica. Five-week-old female BALB/c nude mice were obtained from the Shanghai Institute of Materia Medica (Shanghai, China). Tumor section of A549 xenograft was cut into pieces of around 40 mm^3^ and planted subcutaneously into BALB/c nude mice. Animals were randomly divided into four groups (6 mice per group) to orally receive vehicle (normal saline containing 0.5% CMC-Na), febuxostat (50 mg/kg), 2-DG (400 mg/kg), or with a combination of febuxostat and 2-DG for 28 days. The mice were subjected to fasting from 20:00 pm to 9:00 am every day after drug administration. The investigator was blinded to the group allocation during the experiment. The body weight and tumor volume were measured and recorded twice per week. The tumor volume was calculated as follows: Volume (mm^3^) = 0.5 × length (mm) × width^2^ (mm^2^).

For Western blotting analysis, animals were randomly divided into 4 groups (3 mice per group) when the tumor volumes reached about 100 mm^3^. The mice were administered orally with vehicle, febuxostat at 50 mg/kg, 2-DG at 400 mg/kg or the combination and were fasted for 13 h after dosing. On the next day, the mice were dosed 2 h before sacrifice and the xenografts were collected. Tumor tissues were homogenized in RIPA buffer (Beyotime, Shanghai, China) supplemented with inhibitors of protease and phosphatase (Beyotime, Shanghai, China) and subjected to Western blotting.

### Statistical analysis

All data presented are from at least three independent experiments unless otherwise stated. Statistical analysis was performed using Prism 8 (GraphPad, La Jolla, CA, USA). Differences between two groups were calculated by unpaired two- tailed Student's t-test. Differences among multiple groups were calculated by two-way analysis of variance. Differences were considered statistically significant when the p value was less than 0.05. *: p< 0.05; **: p < 0.01; ***: p < 0.001; ****: p < 0.0001.

## Results

### XDH is highly expressed in LUAD and significantly associated with poor prognosis

**Figure 1:**
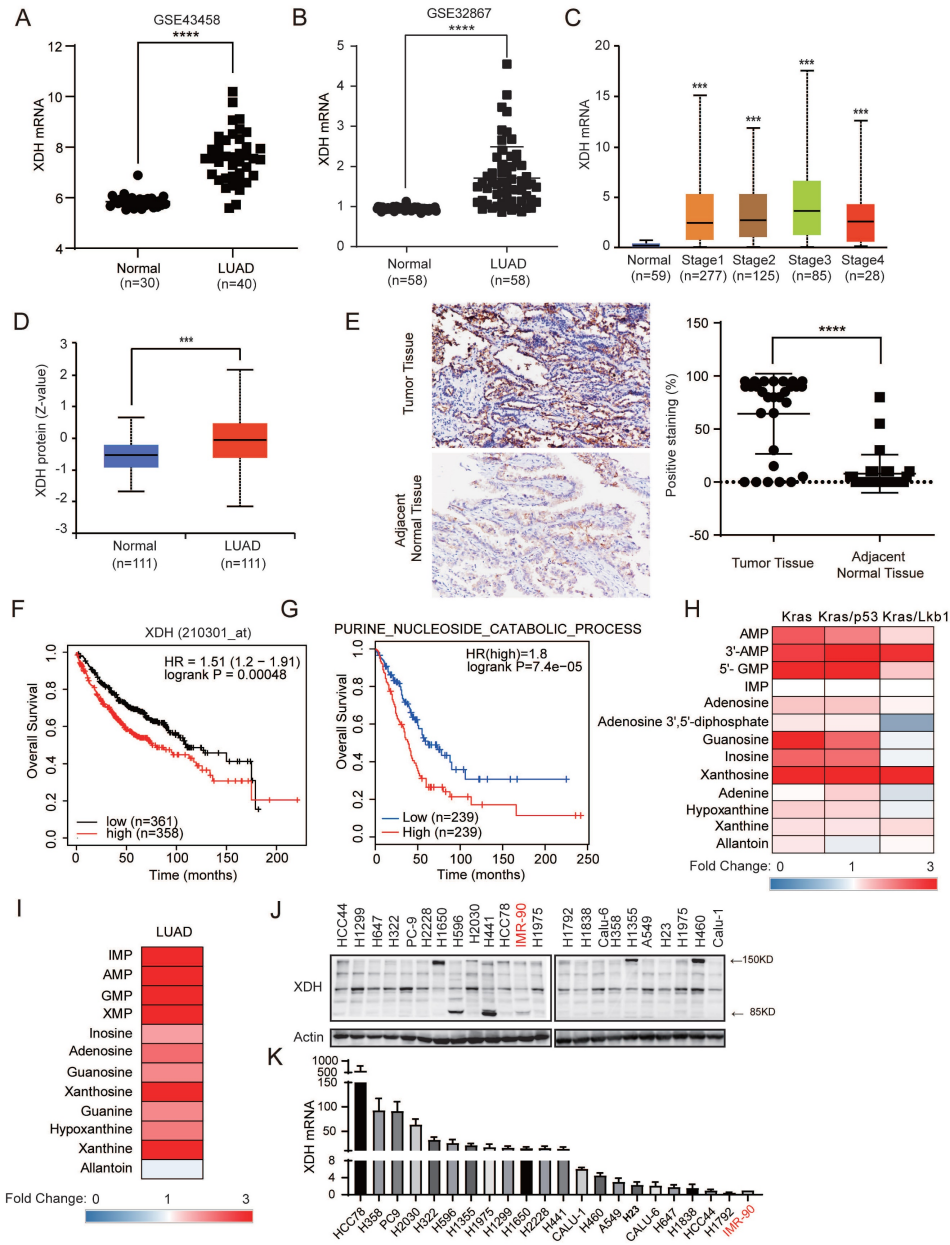
** XDH is highly expressed in LUAD and significantly associated with poor prognosis. (A-B)** The mRNA levels of XDH in normal lung tissues and LUAD tissues were obtained from the Gene Expression Omnibus (GEO) (http://www.ncbi.nlm.nih.gov/geo/) database (GEO accession: GSE43458 and GSE32867). ****: p < 0.0001. **(C)** The expression of XDH according to the different pathologic stage of the patient in the LUAD in the UALCAN database (http://ualcan.path.uab.edu)**.** ***: p< 0.001.** (D)** The protein level of XDH in normal tissue and LUAD. Data was extracted and analyzed using CPTAC (http://ualcan.path.uab.edu/analysis-prot.html). ***: p< 0.001.** (E)** Immunochemistry staining of XDH in LUAD tissues and matched adjacent normal tissues from a cohort of 87 Chinese patients. Representative images were shown. The rate of positive staining in tumor tissue and matched adjacent normal tissue were quantified. **(F)** Overall survival of LUAD patients based on XDH expression according to Kaplan-Meier plotter database (http://kmplot.com/analysis/). **(G)** Kaplan-Meier survival curve in LUAD according to the expression of signature genes involving in purine nucleoside catabolic process was plotted by GEPIA2 database (http://gepia2.cancer-pku.cn/). The gene set of purine nucleoside catabolic process was retrieved from “GOBP_Purine_Nucleoside_Catabolic_Process” in Molecular Signatures Database (MSigDB) (https://www.gsea-msigdb.org/gsea/msigdb/index.jsp). Genes were listed in Supplemental Table S4.** (H)** The level of metabolites in purine degradation in mouse lung tumor driven by Kras, Kras/p53 and Kras/Lkb1 were retrieved from a previous study and the fold changes compared to those in normal lung tissue were plotted as a heatmap. **(I)** The level of metabolites in purine degradation in LUAD were retrieved from a previous study including a cohort of 181 patients and the fold changes compared to those in paired normal adjacent lung tissue were plotted as a heatmap. **(J-K)** The expression of XDH mRNA and protein level in a panel of 21 LUAD cell lines and normal lung fibroblast IMR-90 cells. Data shown are mean ± SD of three independent experiments.

Abnormal XDH expression and activity are observed in various types of cancer and closely associated with the clinical outcome 15. To investigate the XDH expression in LUAD, we retrieved the expression data of XDH in LUAD studies from Gene Expression Omnibus database (GEO) (http://www.ncbi.nlm.nih.gov/geo/) (GSE43458, GSE32867) 34 35. As shown in Fig. 1A & Fig. 1B, the expression of XDH at mRNA level markedly elevated in LUAD tissues compared to normal lung tissues. The expression of XDH was also significantly higher in LUAD at different clinical stages compared to that in normal lung tissues according the data retrieved from UALCAN database (http://ualcan.path.uab.edu) (Fig. 1C), indicating XDH may exert an important role in the development of LUAD. The expression of XDH at protein level was accessed using the Clinical Proteomic Tumor Analysis Consortium 36 (CPTAC, http://ualcan.path.uab.edu/analysis-prot.html). XDH protein was significantly higher in LUAD tumor tissues compared to normal counterparts (Fig. 1D). Consistently, immunohistochemistry analysis of tumor tissues and matched adjacent normal tissues from a cohort of 87 Chinese LUAD patients showed enhanced staining of XDH in LUAD tissues (Fig. 1E). To further study the association between XDH expression and clinical outcome, we analyzed the prognosis of LUAD patients using the Kaplan-Meier plotter database (http://kmplot.com/analysis/) 37. Patients were divided into two groups based on the median level of XDH expression. In this cohort of 719 LUAD patients, 358 patients with elevated XDH expression were revealed to have worse prognosis with a median overall survival time of 75.43 months compared to 110.27 months in patients with low XDH expression (Fig. 1F). Meanwhile, high XDH expression was associated with poor first progression for LUAD (Supplemental Fig. 1A). Moreover, high expression of purine nucleoside catabolic signature genes was significantly correlated with poor prognosis of LUAD (Fig. 1G). Purines biosynthesis provides essential components to promote cancer cell survival and proliferation 6. Enhanced purine nucleoside biosynthetic process was linked to poor prognosis of overall survival in LUAD patients (Supplemental Fig. 1B). We also found that the expression of signature genes representing purine nucleoside biosynthesis and catabolism was positively correlated in LUAD (Supplemental Fig. 1C). We retrieved the data of metabolites involving nucleoside catabolism from the study performed by RJ DeBerardinis's et al. 38, where metabolome was profiled in mice lung tumors driven by oncogenic KRAS, or combined mutations in LKB1 or p53. As shown in Fig. 1H, elevated metabolites in purine degradation were observed in lung tumors driven by Kras, Kras/p53 or Kras/Lkb1. Similarly, by analyzing the data from a large-scale targeted metabolomics study with a cohort of 181 LUAD patients across different histological subtypes 39, we found these metabolites significantly up-regulated in tumor tissue compared to paired normal adjacent tissue (Fig. 1I).

We next examined the expression of XDH at both protein and mRNA levels in a panel of LUAD cell lines and the normal lung fibroblast IMR-90 cells. As shown in Fig. 1J, XDH was detected as intact form at approximately 150 kDa or splicing forms at 85 kDa and 125 kDa, which is consistent with the fact that XDH is a homodimer with each subunit comprising of two Fe2-S2 clusters (20 kDa), one FAD (40 kDa) and one Mo-Co center (85 kDa). Elevated expression of XDH was also observed in LUAD cell lines in comparison to that in normal lung fibroblast IMR-90 cells (Fig. 1J & Fig.1K).

### XDH is required for the survival of starved LUAD cells

**Figure 2:**
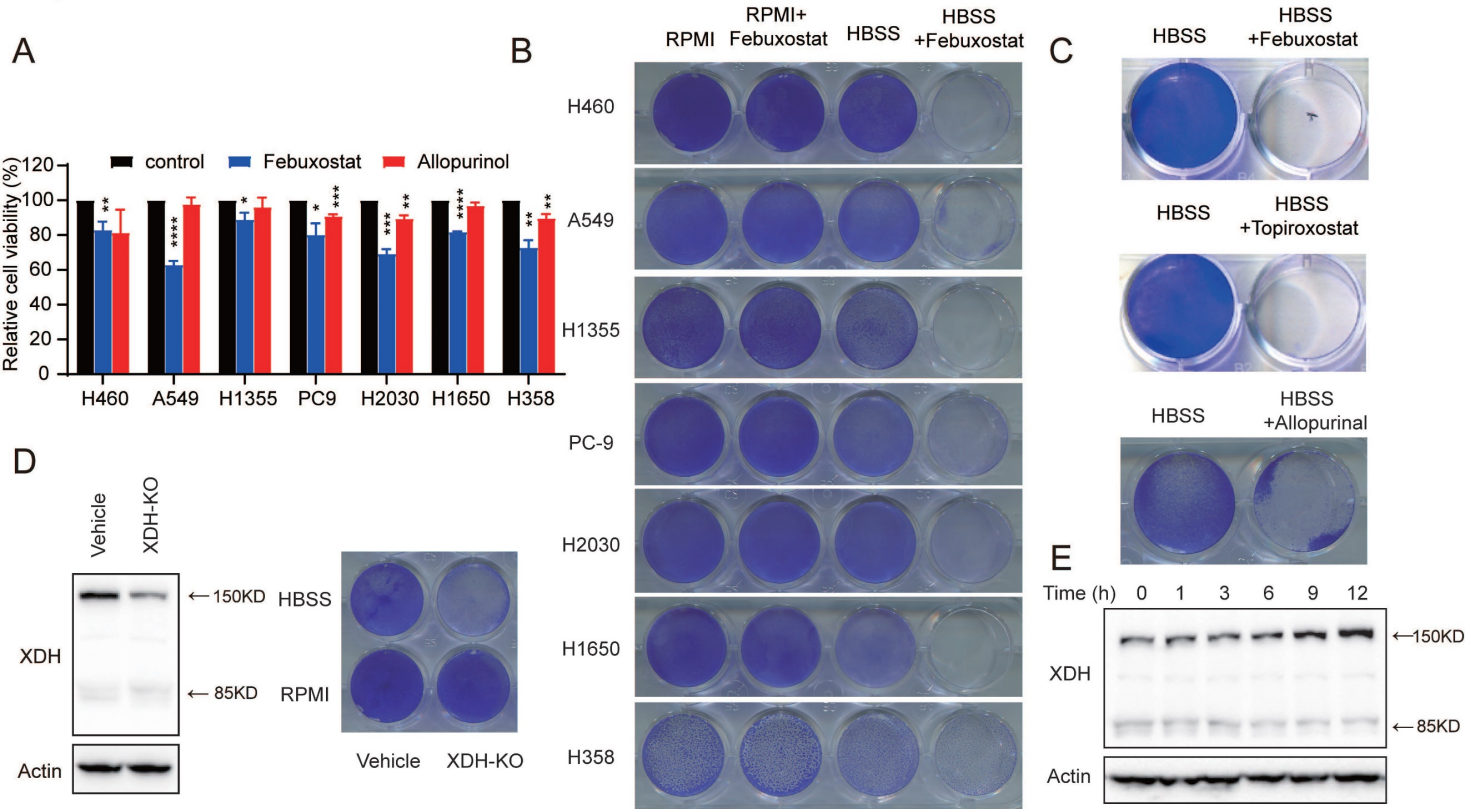
** XDH is required for the survival of starved LUAD cells. (A)** LUAD cells cultured in complete medium were treated with febuxostat (100 μM) or allopurinol (100 μM) for 72 h and cell viability was measured with SRB assay. Data are presented as mean ± SD from three independent experiments.** (B)** Clonogenic survival assay of LUAD cells cultured in complete medium or HBSS in the presence of febuxostat (100 μM). **(C)** Clonogenic survival assay of H460 cells cultured in HBSS containing 100 μM of XDH inhibitors (febuxostat, topiroxostat, and allopurinol). **(D)** XDH-knockout H460 cells were established by CRISPR-Cas9 system. XDH was detected by Western blotting. Clonogenic survival assay was performed in complete medium or HBSS. **(E)** H460 cells were cultured in HBSS for indicated time and XDH was detected by Western blotting. Representative images from three independent experiments were shown.

To explore the potential role of XDH in LUAD, we evaluated the effect of XDH on cell viability. LUAD cells with high expression of XDH at mRNA level (H358, PC9 and H2030), with expression of intact form of XDH (H460, H1650 and H1355) or cells with expression of 85 kDa or 125 kDa spliced forms (A549) were treated with the XDH inhibitor allopurinol or febuxostat. As shown in Fig. 2A, inhibition of XDH moderately attenuated the viability of LUAD cells cultured in complete medium by less than 50%. Nucleotide pools maintained by autophagy were reported to promote the survival of cancer cells in starvation 30, while XDH is a vital and rate-limiting enzyme in purine nucleoside metabolism. We next investigated the role of XDH in response to metabolic stress. LUAD cells were starved by incubated in Hank's balanced salt solution (HBSS) without glucose for 18 h and further cultured in complete medium for 24 h. Cells survived upon the pulsed starvation, while febuxostat attenuated the survival of starved LUAD cells in a concentration-dependent manner and completely abrogated cell survival at 100 μM (Supplemental Fig. 2A). XDH inhibitors was employed at 50, 100 or 200 μM in cellular system in reported studies 40, 41. XDH inhibitors at 100 μM were used in following experiments. As shown in Fig. 2B, Inhibition of XDH by febuxostat significantly attenuated the survival of all tested LUAD cells upon starvation, while it had little effect on the survival of cells continuously incubated in RPMI medium. PI/Annexin V dual staining revealed that inhibition of XDH significantly induced apoptosis in starved LUAD cells (Supplemental Fig. 2B). Two other marketed XDH inhibitors, allopurinol and topiroxostat, were also able to hamper the survival of starved cells (Fig. 2C). We next tested the cytotoxicity of XDH inhibitors on the viability of the immortalized lung fibroblast IMR-90 cells. As shown in Supplemental Fig. 2C & Fig.2D, inhibition of XDH by febuxostat or allopurinol had no effect on the viability of IMR-90 cells in complete medium, while slightly attenuated the survival of IMR-90 cells upon starvation (Supplemental Fig. 2D). To further confirmed the role of XDH in starved cells and ruled out the possible off-target effects of the pharmacological inhibitors, XDH was knocked out in H460 cells. The efficiency of gene editing in XDH was confirmed by significant decreasing in the expression of XDH (Fig. 2D). Similar to XDH inhibitors, decreased XDH expression attenuated cell survival in starvation (Fig. 2D).

Interestingly, we found that XDH was substantially up-regulated following HBSS starvation (Fig. 2E), which suggested that XDH might play an important role in the survival of LUAD cells during starvation. By profiling the frequent mutated genes (KRAS, STK11, KEAP1, TP53, and EGFR) in LUAD cells (https://depmap.org/portal/), we found that the essential role of XDH in starved LUAD cells survival was irrelevant to the frequent somatic mutations in LUAD (Supplemental Fig. 2E). Taken together, these results indicate that XDH is required for the survival of LUAD cells under nutrient stress.

### Nucleosides rescue the survival of starved LUAD cells upon XDH downregulation

**Figure 3:**
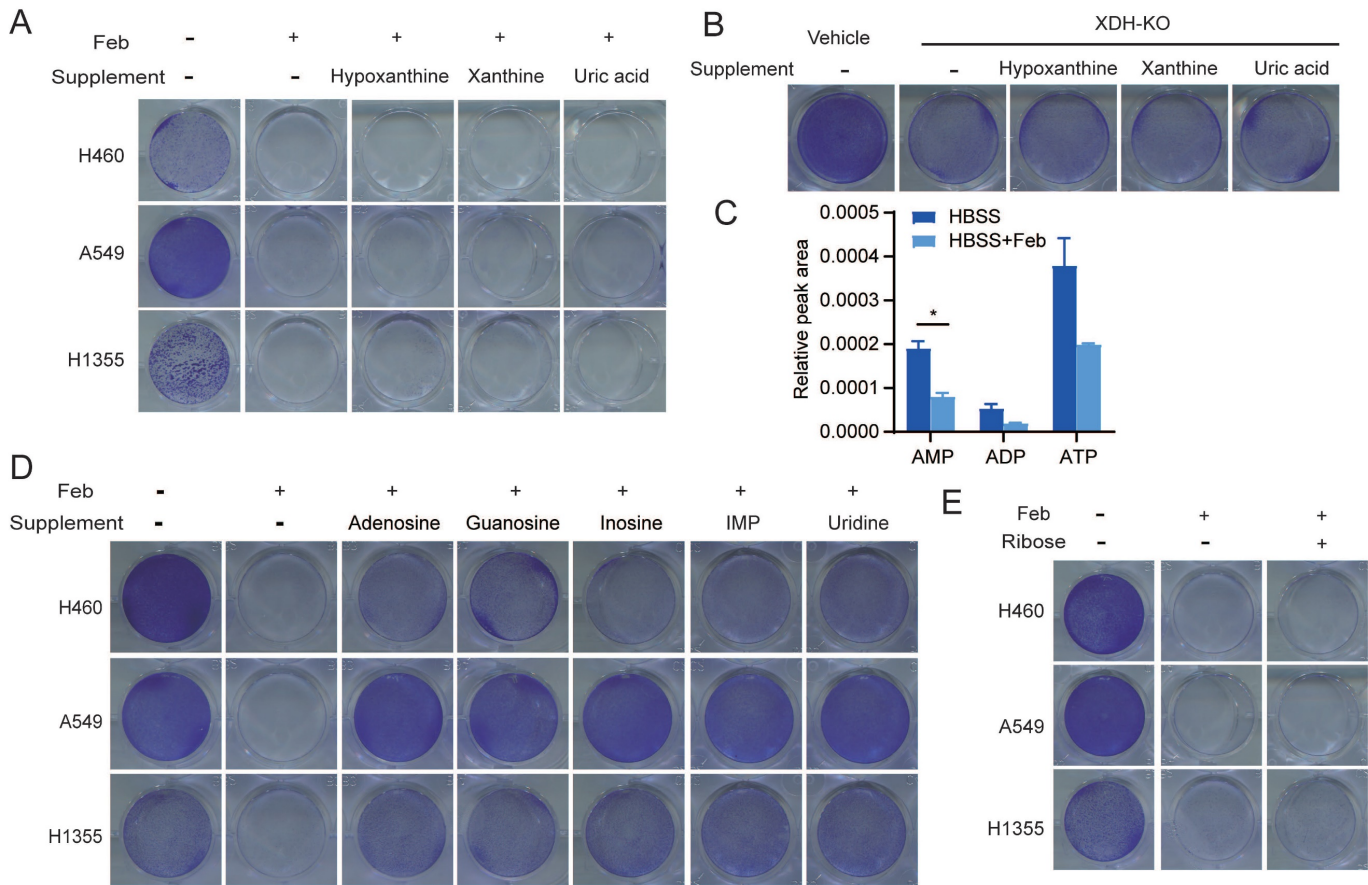
** Nucleosides rescue the survival of starved LUAD cells upon XDH inhibition. (A)** Clonogenic survival assay of LUAD cells in HBSS supplemented with 2 mM of hypoxanthine, xanthine, or uric acid in the presence of febuxostat (100 μM). **(B)** Clonogenic survival assay of XDH-knockout H460 cells in HBSS supplemented with 2 mM of hypoxanthine, xanthine, or uric acid. **(C)** H460 cells cultured in HBSS were treated with febuxostat (100 μM) for 9 h and the levels of adenosine phosphates (AMP, ADP, and AMP) were determined by UPLC-QTOF-MS (n=2). Data shown are mean ± SEM. *: p<0.05. **(D)** Clonogenic survival assay of LUAD cells cultured in HBSS supplemented with 2 mM of adenosine, guanosine, inosine, IMP, or uridine in the presence of febuxostat (100 μM). **(E)** Clonogenic survival assay of LUAD cells cultured in HBSS supplemented with ribose (2 mM) in the presence of febuxostat (100 μM), Representative images from three independent experiments were shown.

To further investigate whether the substrates or products in the enzymatic reaction of XDH are required to mediate the survival of starved cells, LUAD cells were cultured in HBSS supplemented with hypoxanthine, xanthine, or uric acid upon inhibition or knockout of XDH. We found that supplementation with these metabolites couldn't rescue the survival of starved cells with down-regulated XDH (Fig. 3A & Fig. 3B & Supplemental Fig. 3A), indicating that XDH-mediated survival of LUAD cells is not mediated by its substrates or products.

XDH is a critical, rate-limiting enzyme in charge of the last two steps of purine catabolism. Blockade of purine degradation by knockout or inhibiting XDH may affect the level of purine nucleotide as a feedback effect. As shown in Fig. 3C, febuxostat treatment down-regulated the level of ATP, ADP and AMP in starved LUAD cells. Degradation of AMP and GMP produces adenosine and guanosine respectively. Adenosine subsequently deaminated to inosine. Similarly, IMP could metabolize to inosine. Inosine and guanosine can be broken down into hypoxanthine or xanthine, and both produce ribose-1-phosphate in purine catabolism (Supplemental Fig. 3B). Inosine has been reported to sustain T-cells survival via ribose-1-phosphate in the absence of glucose, which fueled into central carbon metabolic pathways 42. We found that supplementation of adenosine, guanosine, inosine or IMP, which were the upstream of ribose-1-phosphate during nucleoside catabolism (Supplemental Fig. 3B), could rescued the survival of starved cells in the presence of febuxostat (Fig. 3D). Similar results were obtained in XDH-knockout LUAD cells cultured in HBSS supplemented with inosine or IMP (Supplemental Fig. 3C). Consistently, supplementation of uridine, which produces ribose by enzymatic degradation (Supplemental Fig. 3D), could also rescue the survival of tested cells (Fig. 3D).

To testify the role of ribose in the survival of starved cells, LUAD cells were cultured in HBSS supplemented with ribose upon inhibition or knockout of XDH. However, ribose failed to rescue cell survival (Fig. 3E & Supplemental Fig. 3E). The conversion to ribose-5-phosphate is required for ribose to be used as a bioenergetics resource, which is mediated by ribokinase and dependent on ATP 43. As XDH inhibition led to a decrease in ATP during starvation (Fig. 3C), the conversion may not be accomplished in this context.

### Inosine-derived ribose fuels key metabolic pathways to sustain the survival of starved LUAD cells

**Figure 4:**
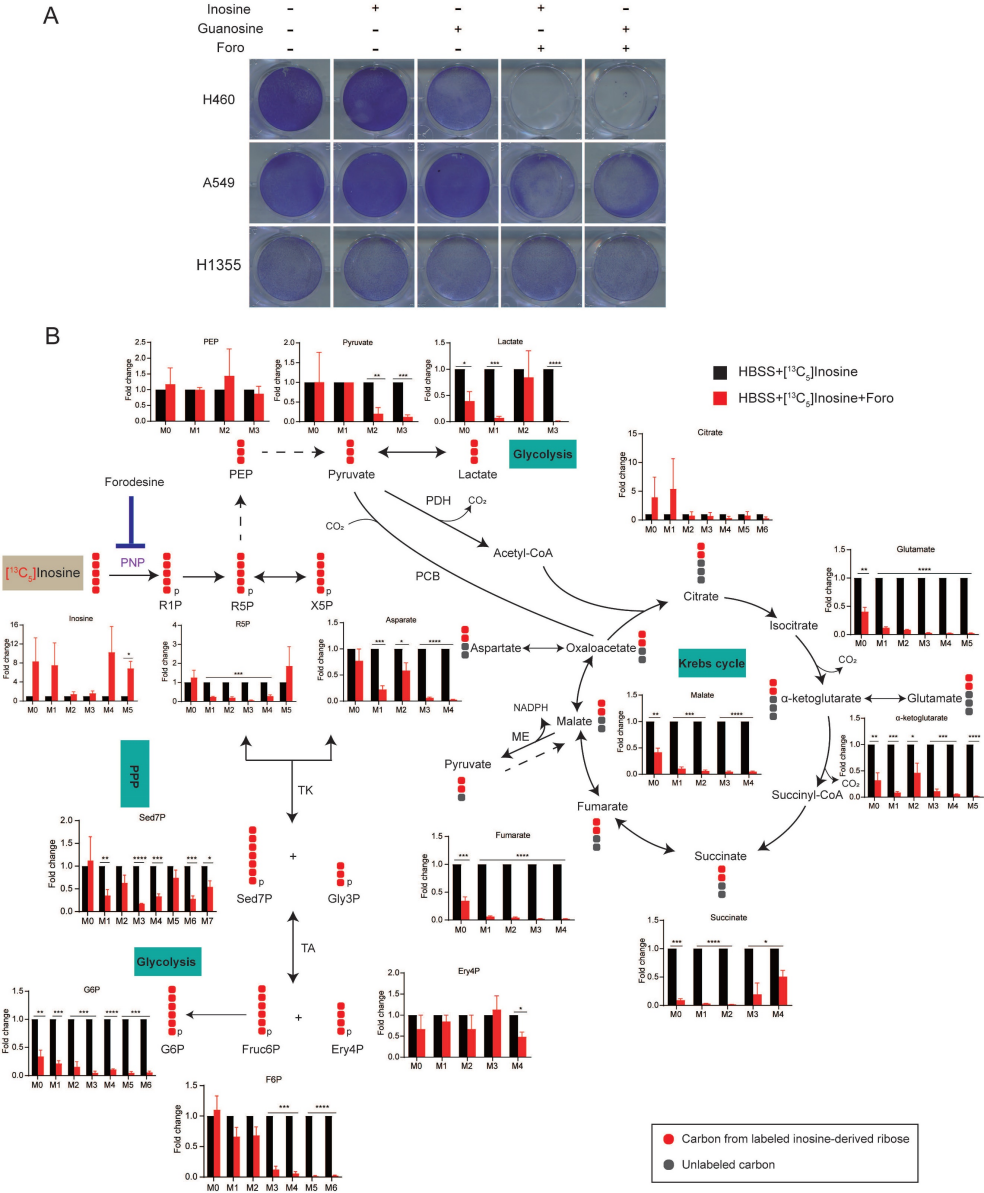
** Inosine-derived ribose fuels key metabolic pathways to sustain the survival of starved LUAD cells. (A)** Clonogenic survival assay of LUAD cells cultured in HBSS supplemented with inosine (2 mM), or Guanosine (2 mM) in the presence of forodesine (10 μM) (n=3). **(B)** H460 cells incubated in HBSS containing [^13^C_5_] inosine (2 mM) were treated with forodesine (10 μM) for 18 h. Cellular metabolites were extracted and analyzed. Numbers on the x axes represent the numbers of ^13^C atoms in the given metabolites. X5P, xylulose-5-phosphate; Gly3P, glyceraldehyde-3-phosphate; PCB, pyruvate carboxylase; ME, malic enzyme; TK, transketolase; TA, transaldolase. Solid or dashed arrow represents single- or multi-step reactions respectively, and single or double-headed arrow refers to irreversible or reversible reactions. Data shown are mean ± SEM (n=3). *: p < 0.05, **: p < 0.01, ***: p < 0.001, ****: p< 0.0001.

Given that nucleosides supplementation rescued the survival of cancer cells, we proposed that ribose derived from nucleoside degradation was important for XDH-mediated cell survival upon starvation. Purine nucleoside phosphorylase (PNP) is responsible for breaking down inosine into ribose-1-phosphate and hypoxanthine (Supplemental Fig. 3B). As shown in Fig. 4A & Supplemental Fig. 4A, inhibition of PNP by forodesine (foro) attenuated the survival of starved LUAD cells supplemented with inosine or guanosine, indicating that ribose derived from nucleoside is essential to cell survival under nutrient stress.

To further confirm that ribose produced by nucleoside is employed as bioenergetic supply and identify its metabolic pathways in starved LUAD cells, we performed isotope tracer studies using [1',2',3',4',5'-^13^C_5_]inosine, in which the ribose contains carbon-13. As shown in Fig. 4B, ribose derived from inosine was metabolized extensively through the central carbon metabolic routes, including the pentose phosphate pathway (PPP), glycolysis and the Krebs cycle. Treatment with foro led to the fractional enrichment of [^13^C_5_] inosine, the metabolic substrate of PNP, and a decrease in the fractional enrichment of the fully ^13^C-labelled species of PPP metabolites, including sedoheptulose-7-phosphate (Sed7P), erythrose-4-phosphate (Ery4P), fructose-6-phosphate (Fruc6P) and glucose-6-phosphate (G6P), which are indirect metabolic products of PNP (Fig. 4B). Moreover, foro treatment greatly reduced the fraction of fully ^13^C isotopologues of glycolysis metabolites, including phosphoenolpyruvate (PEP), pyruvate and lactate, and the fraction of [^13^C_2_] isotopologues of Krebs-cycle metabolites (α-ketoglutarate, glutamate, glutathione, succinate, fumarate, malate and aspartate) (Fig. 4B). These results suggested that the inhibition of PNP suppressed the catabolism of the ribose derived from inosine via the PPP, glycolysis and the Krebs cycle, which may result in reduced energy supply and cell death in starved LUAD cells.

### Down-regulation of XDH impedes UPR and autophagy upon metabolic stress

**Figure 5:**
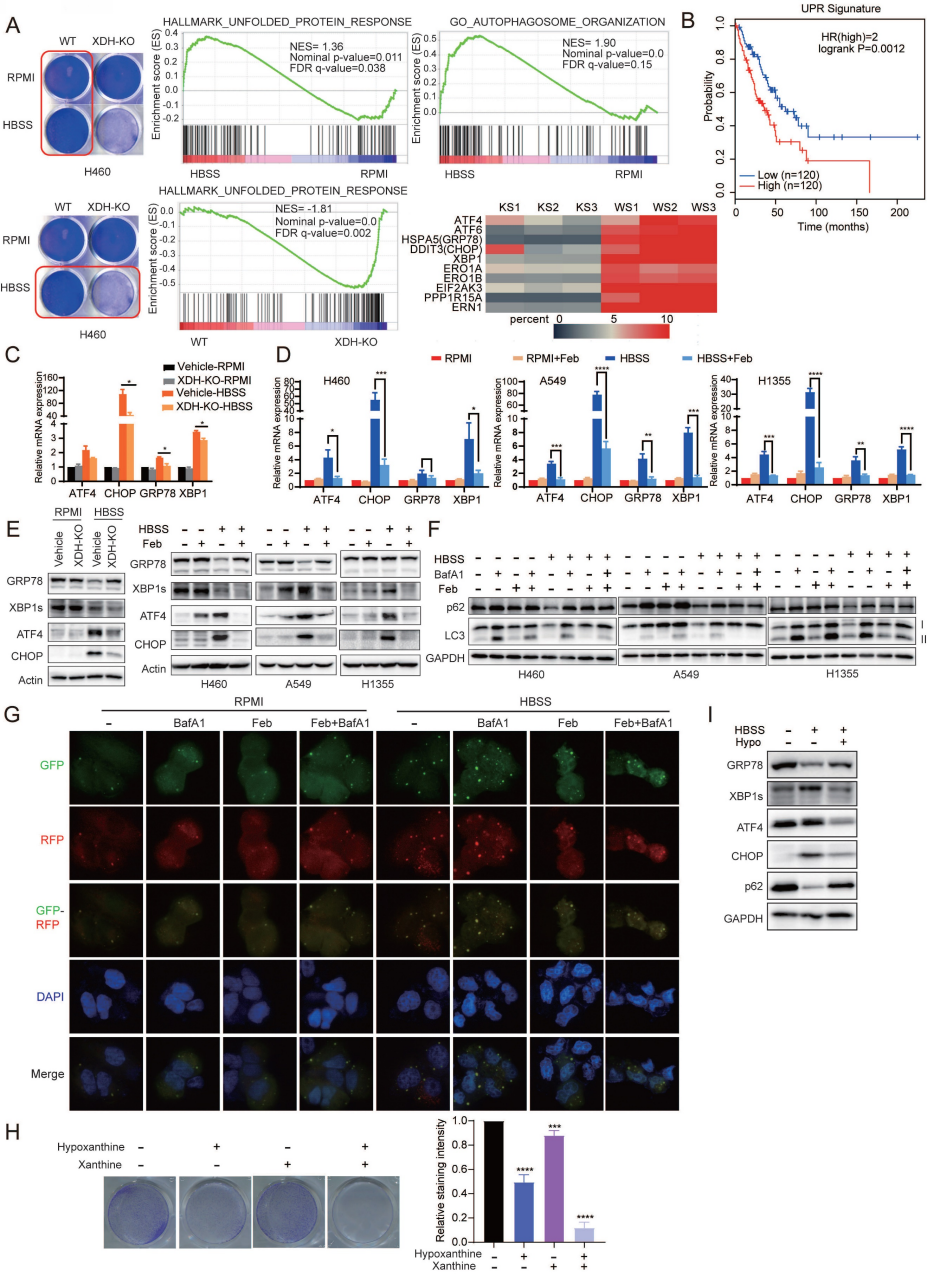
** Down-regulation of XDH impedes UPR and autophagy upon metabolic stress. (A)** Parent or XDH-knockout H460 cells were cultured in complete medium or HBSS for 18 h. Total RNA were extracted and subjected to RNA sequencing (n = 3). GSEA enrichment plots of differentially expressed genes in the gene set of UPR and autophagy signaling pathway were presented. Heatmap of UPR-related gene set was plotted. KS: starved XDH-knockout cells, WS: starved wild type cells. **(B)** The association of overall survival of LUAD patients and the gene signature of UPR was plotted by GEPIA2. The UPR gene set was retrieved from “HALLMARK_Unfolded _Protein_Response” in MSigDB. Genes were listed in Supplemental Table S4. **(C)** XDH-knockout H460 cells were cultured in RPMI or HBSS for 9 h, and the expression of UPR-related genes were detected by RT-qPCR. Data shown were from two independent experiments.** (D)** LUAD cells cultured in RPMI or HBSS were treated with febuxostat (100 μM) for 9 h. mRNA levels of indicated genes were determined by RT-qPCR (n=3).** (E)** The protein levels of UPR-related gene in LUAD cells cultured in RPMI or HBSS upon downregulation (left) or inhibition (right) of XDH. Data shown were from two independent experiments. **(F)** LUAD cells cultured in RPMI or HBSS were treated with febuxostat (100 μM) or baflomycin A1 (50 nM) for 9 h and cell lysates were subjected to Western blotting for the indicated proteins. **(G)** Detection of autophagic flux with the GFP-RFP-LC3 in H460 cells cultured in HBSS containing febuxostat (100 μM) or baflomycin A1 (50 nM) for 9 h. Yellow puncta: autophagosomes (RFP^+^/GFP^+^); red puncta: autolysosomes (RFP^+^/GFP^-^). Representative images from three independent experiments were shown. **(H)** Clonogenic survival assay of H460 cells cultured in HBSS supplemented with 5 mM of hypoxanthine (hypo) or 1 mM of xanthine for 24 h, then cultured in complete medium for additional 24 h. Representative images from three independent experiments were shown. The staining of crystal violet was dissolved in acetic acid and the OD values were measured at 570 nm. Data were presented as mean ± SD. **(I)** H460 cells were cultured in HBSS supplemented with hypoxanthine (10 mM) for 24 h and the cell lysates were subjected to Western blotting for indicated proteins. Data shown were from three independent experiments. **: p < 0.01, ***: p < 0.001, ****: p< 0.0001.

To further explore the mechanism of XDH-mediated cell survival in starved LUAD cells, parent or XDH-knockout H460 cells were incubated in HBSS for 18 h and RNA-seq was performed. Gene Set Enrichment Analysis (GSEA, http://software.broadinstitute.org/gsea/index.jsp) revealed that Unfolded protein response (UPR) and autophagy signaling pathway were activated in starved parental cells (Fig. 5A), which was consistent with the knowledge that UPR and autophagy are usually induced in starved cells in an acute response to nutrient scarcity. However, the gene set of autophagy-related genes failed to be enriched in XDH-knockout cells and the gene set of “Hallmark_Unfolded_Protein_Response” was down-regulated compared that in parental cells (Fig. 5A). The findings suggested that knockout of XDH impeded UPR and autophagy in starved LUAD cells.

Tumor cells are often exposed to harsh microenvironments such as hypoxia or nutrient starvation and trigger UPR 44. Online analysis by GEPIA2 (http://gepia2.cancer-pku.cn/) showed that the high expression of UPR signature was associated with the decreased overall survival in LUAD patients (Fig. 5B). In consistency with the results of transcriptomics, RT-qPCR analysis confirmed that the expression of UPR-related genes including ATF4, CHOP, XBP1 and GRP78 were induced by starvation, while knockout or inhibition of XDH abrogated the induction (Fig. 5C & Fig.5D). Similarly, Induction of XBP1s, ATF4 and CHOP by starvation at protein level were impeded by knockout or inhibition of XDH (Fig. 5E). Interestingly, we observed that the protein level of GRP78 decreased upon starvation, while knockout or inhibition of XDH led to protein accumulation (Fig. 5E & Supplemental Fig.5A). HBSS-induced degradation of GRP78 protein was blocked by the autolysosome inhibitor NH_4_Cl but not the ubiquitin proteasome inhibitor MG132 (Supplemental Fig.5B & Supplemental Fig. 5C), which indicated that autophagy was required for GRP78 degradation. Knockout or inhibition of XDH may impede the process of autophagy and lead to accumulation of GRP78 in starved cells.

UPR is reported to modulate autophagy by transcriptionally regulating a number of ATG genes and subsequently LC3B and Beclin1 45. Autophagy is a well-organized process of intracellular degradation to generate amino acids, nucleotides and fatty acids that are recycled into metabolic and biosynthetic pathways. The process is rapidly activated under starvation and buffers metabolic stress 46. As shown in Fig. 5F, starvation of LUAD cells resulted in reduced levels of p62, LC3-I and LC3-II, indicating the induction of a high rate of autophagic flux as described previously 47. Accumulation of p62 and LC3-II were observed in starved cells treated with baflomycin A1 (BafA1) (Fig. 5F), which inhibits the fusion between autophagosomes and lysosomes as well as autophagic degradation. However, inhibition of XDH resulted in the accumulation of p62 and unprocessed LC3-I under nutrient stress even in the presence of baflomycin A1 (Fig. 5F), indicating the attenuation of the onset of autophagic process in starved LUAD cells. We further examined autophagic flux using the GFP-RFP-LC3 assay. Due to quenching of the GFP fluorescence in acidified compartments, yellow puncta (positive for both GFP and RFP signals) represent autophagosomes, while red-only puncta indicate autolysosomes. Upon HBSS starvation, a number of red-only puncta were detected in starved H460 cells, while red and yellow LC3 puncta significantly decreased in XDH inhibitors-treated cells (Fig. 5G), demonstrating the blockade of autophagy.

To further confirm the role of XDH in modulating UPR and autophagy induced by starvation, LUAD cells were cultured in HBSS supplemented with excessive substrates of XDH, i.e. hypoxanthine or xanthine, to mimic XDH inhibition. In agreement with down-regulation of XDH, supplementation of hypoxanthine or/and xanthine promoted cell death in starvation (Fig. 5H), which was accompanied with blockade of UPR and autophagy (Fig. 5I).

### Down-regulation of XDH attenuates amino acids recycling and cell survival signaling in metabolic stress

We found that down-regulation of XDH attenuated the autophagy in starved LUAD cells, which was consistent with the blockade of nucleoside catabolism upon XDH inhibition (Fig. 3C & Fig.3D). Autophagy is critical in sustaining amino acid levels to meet cellular demand when cells are deprived of nutrients 30, 48. mTORC1 is a master growth regulator that senses nutrients cues, including amino acids 49.

**Figure 6:**
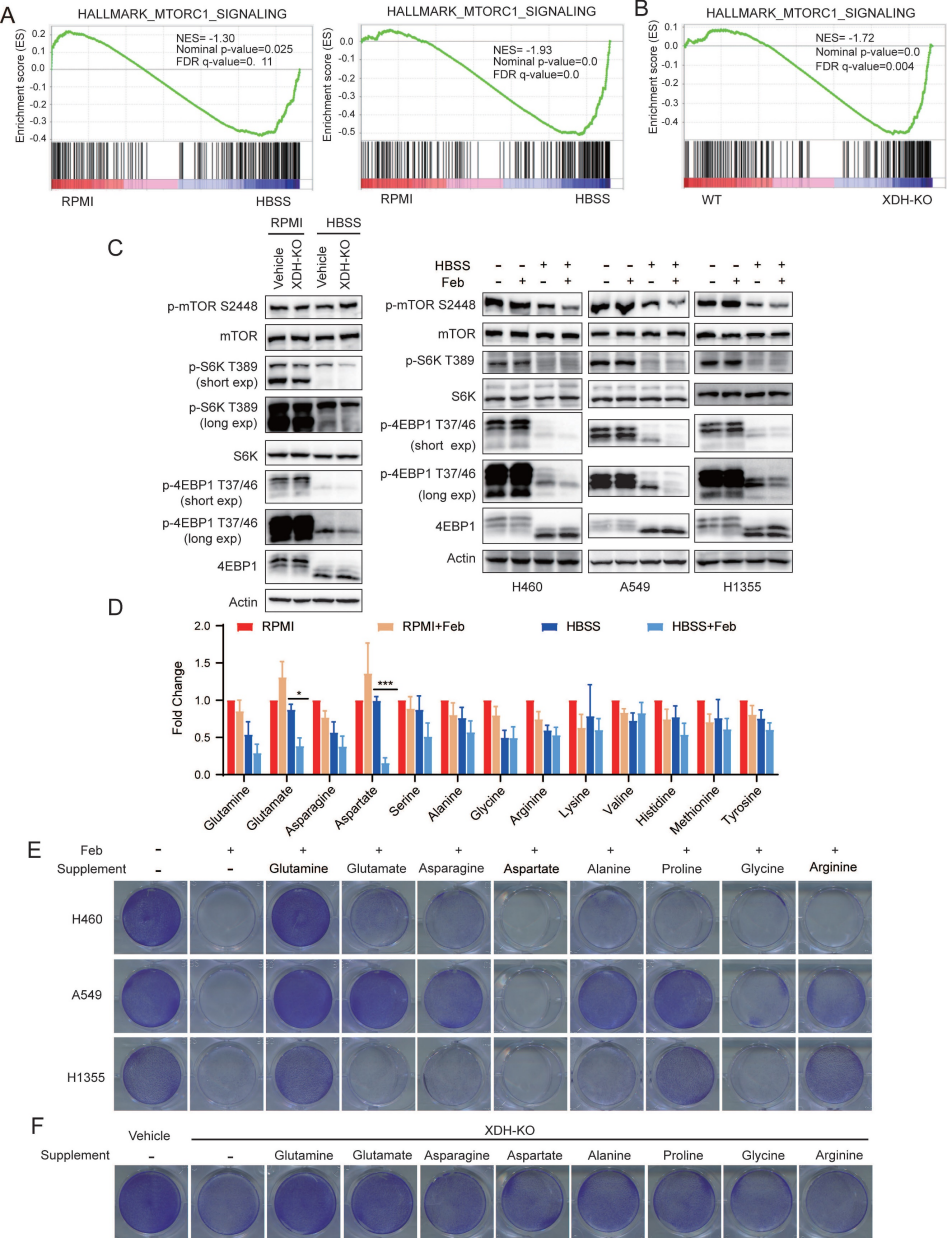
** Down-regulation of XDH attenuates amino acids recycling and cell survival signaling in metabolic stress. (A)** GSEA enrichment plot of the gene set of mTORC1 signaling in wild type (left) or XDH-knockout (right) H460 cells upon starvation.** (B)** GSEA enrichment plot of the gene set of mTORC1 signaling in starved XDH-knockout vs wild type H460 cells. **(C)** XDH-knockout H460 cells or LUAD cells incubated with febuxostat (100 μM) were cultured in RPMI or HBSS for 9 h and indicated proteins were detected by Western blotting. **(D)** H460 cells cultured in RPMI or HBSS were treated with febuxostat (100 μM) for 9 h and intracellular levels of amino acids were determined. Data shown are mean ± SEM from three independent experiments (normalized to cell number).** (E)** Clonogenic survival assay of LUAD cells cultured in HBSS supplemented with 2 mM of glutamine, glutamate, asparagine, aspartate, alanine, proline, glycine or arginine in the presence of febuxostat (100 μM). Representative images from three independent experiments were shown. **(F)** Clonogenic survival assay of XDH-knockout H460 cells in HBSS supplemented with 2 mM of glutamine, glutamate, asparagine, aspartate, alanine, proline, glycine or arginine. Representative images from three independent experiments were shown.

mTORC1 signaling was down-regulated in both wild type and XDH-knockout H460 cells upon nutrient deprivation (Fig. 6A), which was consistent with the role of mTORC1 as a negative regulator of autophagy. Notably, amino acids derived from the autophagic degradation would re-activate mTORC1 in starved cells 50, 51. Transcriptomic analysis revealed that mTORC1 signaling was significantly down-regulated in starved XDH-knockout cells compared to that in wild type cells, which was in agreements with the blockade of autophagic degradation in starved LUAD cells upon XDH downregulation (Fig. 6B). Consistently, phosphorylated mTOR as well as its substrates ribosomal protein S6 kinase (S6K) and translation initiation factor 4E binding protein 1 (4EBP1) decreased upon HBSS starvation, which further reduced in XDH-knockout H460 cells (Fig. 6C, left panel). Similar observation was obtained in starved LUAD cells treated with febuxostat (Fig. 6C, right panel).

To further investigate the effect of XDH on the protein degradation during starvation, we examined the level of amino acids in starved H460 cells treated with febuxostat. As shown in Fig.6D, XDH inhibition further decreased the level of most amino acids in starved cells, which was in reconciled with the decreased mTORC1 signaling (Fig. 6A & Fig.6B). In particular, glutamate and aspartate which are associated with the process of TCA cycle and nucleotide biosynthesis significantly diminished among the detected amino acids (Fig. 6D). To elucidate the role of amino acids in the survival of starved LUAD cells mediated by XDH, different types of amino acids were supplemented in HBSS respectively in the presence of febuxostat. As shown in Fig.6E, supplementation of glutamine, glutamate or other amino acids rescued the survival of starved LUAD cells. Glutamine and glutamate supplementation also rescued the survival of XDH-knockout cells upon starvation (Fig. 6F & Supplemental Fig. 6A). Though degradative processes other than autophagy may also breakdown intracellular macromolecules upon starvation, these results demonstrated that amino acids recycling by autophagic degradation were important for XDH-mediated LUAD cells survival under nutrient stress.

### XDH inhibitor potentiates the anti-proliferative activity of 2-deoxy-D-glucose in LUAD cells

**Figure 7:**
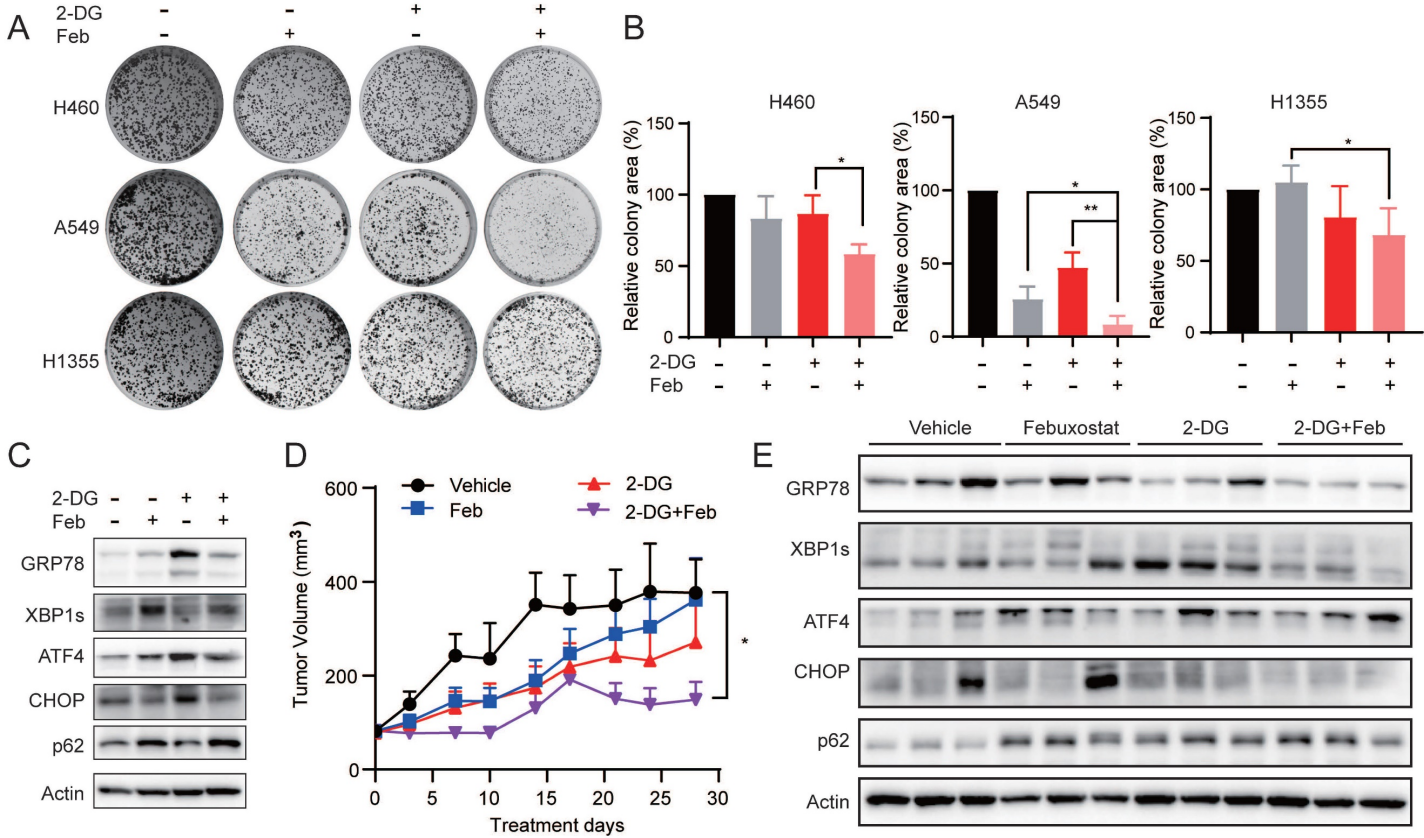
** XDH inhibitor potentiates the anti-proliferative activity of 2-deoxy-D-glucose in LUAD cells. (A)** Colony formation LUAD cells treated with 2-DG (1 mM) or febuxostat (100 μM) alone or in combination. Representative images from three independent experiments were shown. **(B)** Quantitation of colony areas with ImageJ. *: p < 0.05, **: p < 0.01. **(C)** A549 cells were pre-treated with febuxostat (100 μM) for 12 h, then concurrently with 2-DG (1 mM) for additional 24 h. Cell lysates were subjected to Western blotting for the indicated proteins. **(D)** Randomly grouped mice bearing A549 xenografts were administrated orally with vehicle control, 2-DG (400 mg/kg, once a day), febuxostat (50 mg/kg, once a day), or the combination of 2-DG and febuxostat. Mice were fasted for 13 h after each administration. Tumor volume was measured twice a week. Data were presented as mean ± SEM. Differences between indicated groups were analyzed using two-way ANOVA. *: p < 0.05. **(E)** Mice bearing A549 cell-derived xenografts were administered twice as described in **(D)**. Tumor tissue were collected 2 h after the last dosing and subjected to Western blotting for indicated proteins.

Given that the down-regulation of XDH might induce cells death in starved LUAD cells by impending UPR and autophagic degradation, we sought to explore whether targeting XDH would enhance the antitumor activity of drugs that are able to induce UPR or autophagy. 2-deoxy-D-glucose (2-DG) is an inhibitor of glycolysis and has been reported to induce UPR and autophagy 52, 53. As shown in Fig. 7A & Fig.7B, febuxostat significantly enhanced the activity of 2-DG to inhibit the clonogenic growth in H460, A549 and H1355 cells. Consistently, 2-DG treatment induced the expression of UPR-related protein GPR78, ATF4 and CHOP, as well as the degradation of p62, while febuxostat treatment impeded the process (Fig. 7C).

To evaluate the effect of febuxostat on the efficacy of 2-DG in vivo, mice bearing A549 xenograft orally received febuxostat or 2-DG alone or their combination. Febuxostat administration significantly reduced the level of uric acid in the plasma of mice (Supplemental Fig. 7A), indicating the inhibition of XDH. Febuxostat or 2-DG alone displayed little effect on the tumor growth, while the combinatorial treatment significantly potentiated the activity of 2-DG with a T/C value of 42.01% (Fig. 7D). Moreover, no significant differences in body weight were observed among the groups (Supplemental Fig.7B). Brief treatment of 2-DG induced UPR response in A549 tumor demonstrated as induction of GRP78, XBP1s and CHOP, while concurrent treatment of febuxostat circumvented the process (Fig. 7E). These results indicated that febuxostat potentiated the activity of 2-DG against LUAD by suppressing the induction of UPR.

## Discussion

Increasing evidences indicate that XDH is associated with the progression of cancer, while the insight mechanism remains elusive. In this study, we for the first time revealed that XDH was essential to mediate the survival of LUAD cells under nutrient stress. Knockout or inhibition of XDH induced cell death in starved LUAD cells by blocking UPR and autophagy, which generated amino acids and nucleotides to maintain the cell survival. Inhibition of XDH potentiated the activity of 2-DG that induced UPR and/or autophagy against LUAD in vitro and in vivo.

XDH activity significantly increased in multiple cancer types including bladder cancer 54, small cell and non-small cell lung cancer 55. Consistently, we found higher expression level of XDH in LUAD tissues and cell lines, which was negatively associated with the prognosis of LUAD patients. However, XDH inhibitors possessed moderate activity to inhibit cell viability of LUAD cells cultured in the complete medium. Similarly, it has been reported that inhibition of XDH by allopurinol or knockdown XDH by siRNA displayed mild anti-proliferative activity even in “sensitive” LUAD cells 56. Interestingly, we found that XDH was required for the survival of starved LUAD cells evidenced by cell death induced by XDH inhibition or down-regulated XDH expression. The enzymatic activity of XDH significantly increased under mechanical stress 57. Induction of XDH was also found in LUAD cells under nutrient stress, further indicating the important role of XDH in response to starvation. XDH regulated cancer development mostly by the action of its catalytic products such as uric acid and ROS 20, 58. However, supplementation of either the substrates (hypoxanthine or xanthine), or the product (uric acid) failed to rescue the starved LUAD cells treated with febuxostat. We found that XDH might facilitate the degradation of nucleotide to provide nutrients for cell survival. Inhibition of XDH down-regulated the level of ATP, ADP and AMP in starved LUAD cells, while supplementation of adenosine, guanosine, inosine or IMP that sits upstream of ribose-1-phosphate during nucleoside catabolism rescued the survival of starved cells treated with febuxostat. In particular, inosine-derived ribose was revealed to fuel key metabolic pathways to sustain the survival of starved LUAD cells, while blockade of inosine degradation by inhibiting PNP attenuated the ribose-mediated metabolism as well as the survival of starved LUAD cells. Our findings are consistent with the previous report that ribose-derived inosine sustained the proliferation of T-cells and cancer cells under glucose restriction by providing metabolic energy and biosynthetic precursors via glycolysis and the PPP 42. Thus, blockade of the final step of purine degradation catalyzed by XDH might attenuate the degradation of nucleotide possibly as a feedback effect, which deserved further investigation.

UPR and autophagy are usually activated to promote cell survival during nutrient stress 59, 60. Indeed, UPR and autophagy were induced in LUAD cells upon starvation by RNA-seq analysis and profiling key proteins in the process. Strikingly, we found the final step of purine degradation catalyzed by XDH was important for the process of UPR and autophagy. Knockout or inhibition of XDH abrogated the induction of UPR and attenuated the autophagy flux. Moreover, incubation of LUAD cells in HBSS supplemented with excessive substrates to mimic XDH inhibition blocked UPR and autophagy and promoted cell death. Autophagy promotes the survival of cancer cells by alleviating metabolic stress and providing nutrients, including amino acids, nucleosides, fatty acids and sugars via the degradation of cellular organelles and unfolded proteins 61. Therefore, the aforementioned blockade of nucleotide degradation by XDH inhibition might be also due to suppression of autophagy. Consistently, the levels of amino acids especially aspartate and glutamate were further declined accompanied with inactivated mTORC1 signaling in starved LUAD cells upon XDH-inhibition. Supplementation of amino acids rescued the survival of starved LUAD cells upon inhibition or knockout of XDH. Similar results have been reported in autophagy-deficient lung cancer cells 30. The level of amino acids and nucleosides significantly decreased in Atg7-deficient lung cancer cells compared to that in autophagy-competent cells cultured in HBSS, while supplementation of glutamine, glutamate or nucleosides rescued the survival of Atg7-deficient lung cancer cells 30. Collectively, these findings indicated XDH may play a key role in sustaining the survival of starved LUAD cell by facilitating UPR and autophagy. Meanwhile, XDH may also modulate other biological processes to maintain cell survival, which deserves further investigation.

UPR or autophagy has currently been identified as an important process to mediate cells survival and to circumvent the efficacy of chemotherapy as well as targeted therapy 45, 62. We found that XDH inhibitors potentiated the anti-cancer activity of 2-DG, which was accompanied with abrogation of 2-DG-induced UPR. This finding further confirmed the critical role of XDH in modulating UPR/autophagy and also suggested that inhibiting XDH would improve the efficacy of drugs capable of inducing UPR/autophagy. In fact, autophagy inhibitors such as CQ or HCQ have been combined with chemotherapy or targeted drugs for the treatment of multiple types of cancer, including colorectal cancer, breast cancer, HCC and recurrent non-small cell lung cancer 62. However, the retinal toxicity of CQ and HCQ should be concerned in clinic 63. The dose of 2-DG was limited by its toxicity associated with hypoglycemia symptoms 64, while its combination with the XDH inhibitor febuxostat was well tolerated in this study. As XDH inhibitors have been used for the therapy of gout for a long time, it is worthwhile to for further investigate the efficacy of XDH inhibitors in combination of drugs inducing UPR/autophagy. For example, the glutaminolysis inhibitor was found to induce pro-survival autophagy via activating ATF4, which compromised its efficacy against colorectal cancer 65. It would be of great interest to evaluate whether inhibition of XDH would enhance the activity of glutaminolysis inhibitors in LUAD. However, the safety of that combination of XDH inhibitors with anti-cancer drugs inducing UPR/autophagy should be systematically studied before the clinical trial.

**Figure 8:**
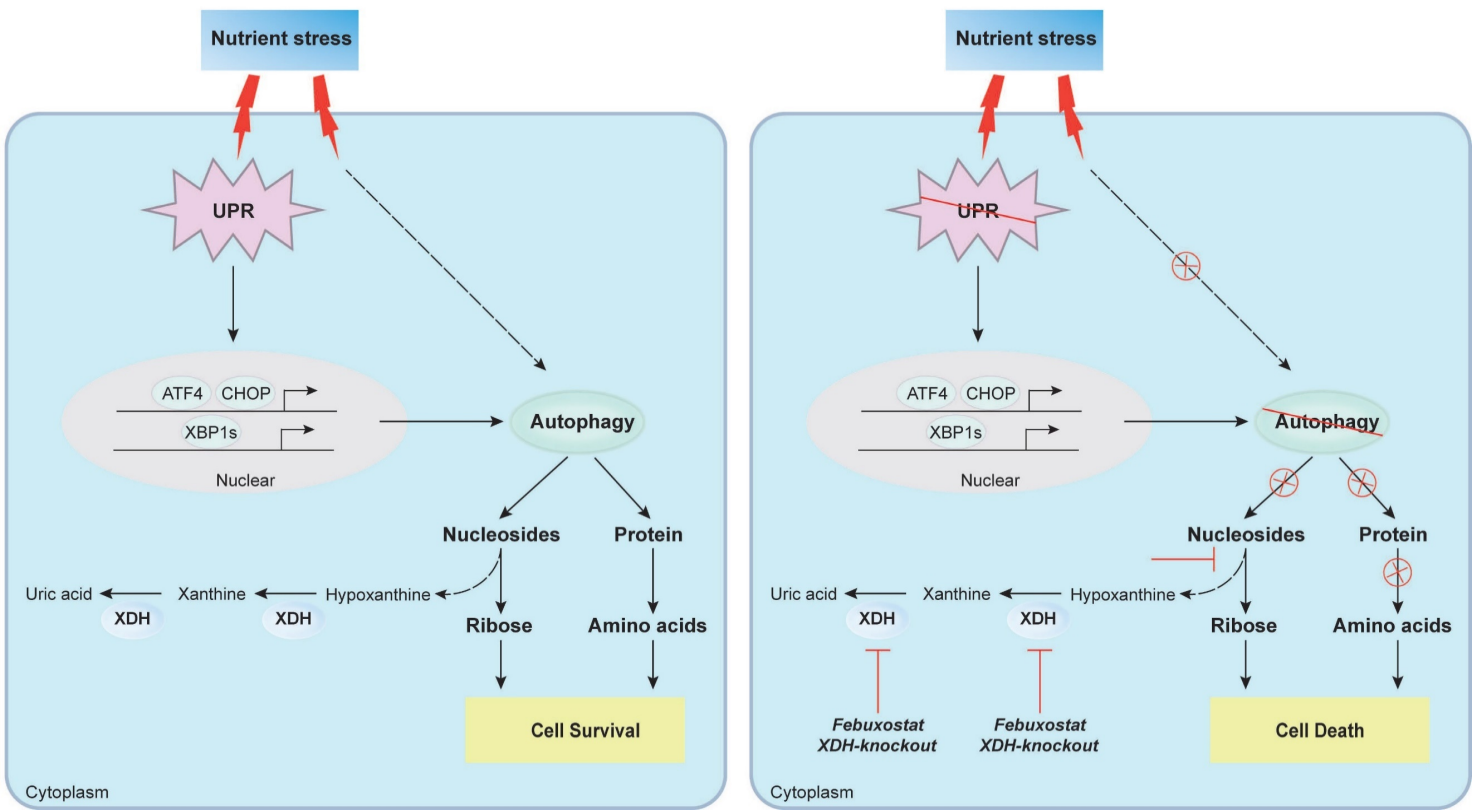
** Proposed mechanism of XDH-mediated LUAD cell survival under nutrient stress.** XDH supported the survival of starved LUAD cells by facilitating UPR and autophagy. Conversely, when XDH was downregulated or inhibited, the starved LUAD cells undergo death due to blockade of the degradation of nucleosides and proteins via UPR and autophagy.

In summary, we found that XDH was required for the survival of LUAD cells under nutrient stress by facilitating UPR and autophagy (Fig.8). Knockout or inhibition of XDH attenuated UPR and autophagy in starved LUAD cells, which resulted in nutrients scarcity due to blockade of the degradation of nucleosides and proteins, and finally cell death (Fig.8). These findings support the notion that targeting XDH may be a novel strategy to improve the efficacy of anti-tumor drugs that induce UPR and autophagy for the therapy of LUAD.

## Supplementary Material

Supplementary figures and tables.
