# Supplementary material for: Xanthine dehydrogenase rewires metabolism and the survival of nutrient deprived lung adenocarcinoma cells by facilitating UPR and autophagic degradation
Source: Int J Biol Sci. 2023 Jan 1;19(3):772–88. doi: 10.7150/ijbs.78948 (PMC9909990; doi:10.7150/ijbs.78948)
Supplement: Supplementary file 1 — Supplementary figures and tables. [file ijbsv19p0772s1.pdf]

Supplemental materials

Supplemental Tables

Table S1. The formulation of Hank's balanced salt solution (HBSS)

Ingredient	Concentration (mg/L)
Calcium Chloride ($CaCl_2$) (anhydrous)	140
Magnesium Chloride ($MgCl_2 \cdot 6H_2O$)	100
Magnesium Sulfate ($MgSO_4 \cdot 7H_2O$)	100
Potassium Chloride (KCl)	400
Potassium Phosphate monobasic (KH_2PO_4)	60
Sodium Bicarbonate ($NaHCO_3$)	350
Sodium Chloride (NaCl)	8000
Sodium Phosphate dibasic (Na_2HPO_4)	48

Table S2. Primers used for qPCR

**Gene name (Forward-F and Reverse-R)**	Primer sequences
XDH-F	5'-AACCATCTCAGCCCTCAAGA-3'
XDH-R	5'-AGCTCCTCCTTCCAGAGCTT-3'
HSPA5 (GRP78)-F	5'- CATCACGCCGTCCTATGTCG-3'
HSPA5 (GRP78)-R	5'- CGTCAAAGACCGTGTTCTCG-3'
DDIT3 (CHOP)-F	5'- GGAAACAGAGTGGTCATTCCC-3'
DDIT3 (CHOP)-R	5'-CTGCTTGAGCCGTTCATTCTC-3'
XBP1-F	5'-CCGCAGCAGGTGCAGG-3'
XBP1-R	5'-GAGTCAATACCGCCAGAATCCA-3'
ATF4-F	5'-CCCTTCACCTTCTTACAACCT-3'
ATF4-R	5'-TGCCCAGCTCTAAACTAAAGGA-3'
Actin-F	5'-CATGTACGTTGCTATCCAGGC-3'
Actin-R	5'- CTCCTTAATGTCACGCACGAT-3'
18S-F	5'-GCGGCGGAAAATAGCCTTTG-3'

18S-R	5'-GATCACACGTTCCACCTCATC-3'

Table S3. sgRNA oligonucleotides used to construct XDH-knockout H460 cells

sgXDH #1	Oligo1	CACCGCGTGTTCCCCACGACCAGCT
	Oligo2	AAACAGCTGGTCGTGGGGAACACGC
sgXDH #2	Oligo1	CACCGCTCTAGGATGGTGGATGCTG
	Oligo2	AAACCAGCATCCACCATCCTAGAGC

Table S4. Lists of signature genes included in the respective gene sets.

Purine nucleoside catabolic process	ADA
	ADAL
	ENPP4
	GDA
	NUDT1
	PNP
	XDH
Purine nucleoside biosynthetic process	ADA
	ADAL
	ADK
	AMD1
	APRT
	HPRT1
	MTAP
	NT5E
	PNP
	PRTFDC1
Unfolded protein response	ALDH18A1
	ARFGAP1
	ASNS
	ATF3
	ATF4
	ATF6
	ATP6V0D1
	BAG3
	BANF1
	CALR
	CCL2
	CEBPB
	CEBPG
	CHAC1
	CKS1B
	CNOT2

	CNOT4
	CNOT6
	CXXC1
	DCP1A
	DCP2
	DCTN1
	DDIT4
	DDX10
	DKC1
	DNAJA4
	DNAJB9
	DNAJC3
	EDC4
	EDEM1
	EEF2
	EIF2AK3
	EIF2S1
	EIF4A1
	EIF4A2
	EIF4A3
	EIF4E
	EIF4EBP1
	EIF4G1
	ERN1
	EXOC2
	EXOSC1
	EXOSC10
	EXOSC2
	EXOSC4
	EXOSC5
	EXOSC9
	FKBP14
	FUS
	GEMIN4
	GOSR2
	H2AX
	HERPUD1
	HSP90B1
	HSPA5
	HSPA9
	HYOU1
	IARS1
	IFIT1
	IGFBP1

	IMP3
	KDELR3
	KHSRP
	KIF5B
	LSM1
	LSM4
	MTHFD2
	NABP1
	NFYA
	NFYB
	NHP2
	NOLC1
	NOP14
	NOP56
	NPM1
	PAIP1
	PARN
	PDIA5
	PDIA6
	POP4
	PREB
	PSAT1
	RPS14
	RRP9
	SDAD1
	SEC11A
	SEC31A
	SERP1
	SHC1
	SLC1A4
	SLC30A5
	SLC7A5
	SPCS1
	SPCS3
	SRPRB
	SSR1
	STC2
	TATDN2
	TSPYL2
	TTC37
	TUBB2A
	VEGFA
	WFS1
	WIPI1

	XBP1
	XPOT
	YIF1A
	YWHAZ
	ZBTB17

Supplemental methods

Apoptosis assay

Cell apoptosis was measured with an annexin V–FITC apoptosis detection kit (KeyGEN BioTECH) according to the manufacturer's protocol. Briefly, LUAD cells cultured in complete medium (RPMI 1640 with 10% FBS) were seeded in 6-well plates. When cell density reached about 80% confluent, cells were washed with PBS twice and further incubated in RPMI or HBSS and exposed to febuxostat (100 μM) for 18 h. Cells were collected and subjected to dual staining of annexin V–FITC and propidium iodide (PI). Cells undergoing apoptosis were measured with a FACS Calibur flow cytometer (BD Biosciences) and data were analyzed with FlowJo software.

Quantification of uric acid in blood plasma

Mice bearing A549 xenografts treated with febuxostat and 2-DG alone or in combination for 28 days. Blood was collected 2 h post the last dosing and plasma was separated by centrifugation at 3000 rpm for 10 min at 4 °C. The concentration of uric acid in the plasma was detected with a uric acid Test Kit (Nanjing Jiancheng Bioengineering Institute, Nanjing, China) according to the manufacturer's instructions.

Supplemental Figures

Supplemental Figure 1

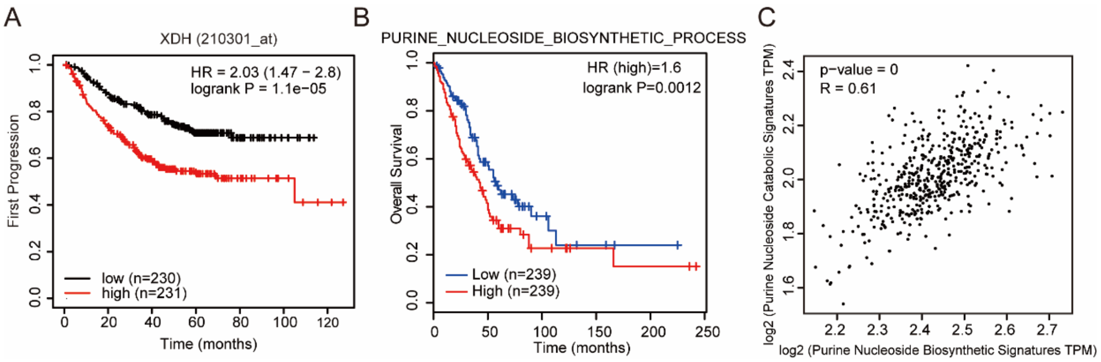

Supplementary Figure 1. Dysregulation of XDH and purine metabolism in LUAD were associated with poor clinical outcome.

(A) The Kaplan–Meier curve of first progression in LUAD patients based on XDH expression. (B) Kaplan-Meier survival curve in LUAD according to the expression of signature genes involving in purine nucleoside biosynthesis process was plotted by GEPIA2 database (http://gepia2.cancer-pku.cn/). The gene set of purine nucleoside catabolic process was retrieved from "GOBP_Purine_Nucleoside_Biosynthetic_Process" in MSigDB. Genes were listed in Supplemental Table S4. (C) The correlation of the signature genes in purine nucleoside biosynthesis and catabolism in LUAD was plotted by GEPIA2.

Supplemental Figure 2

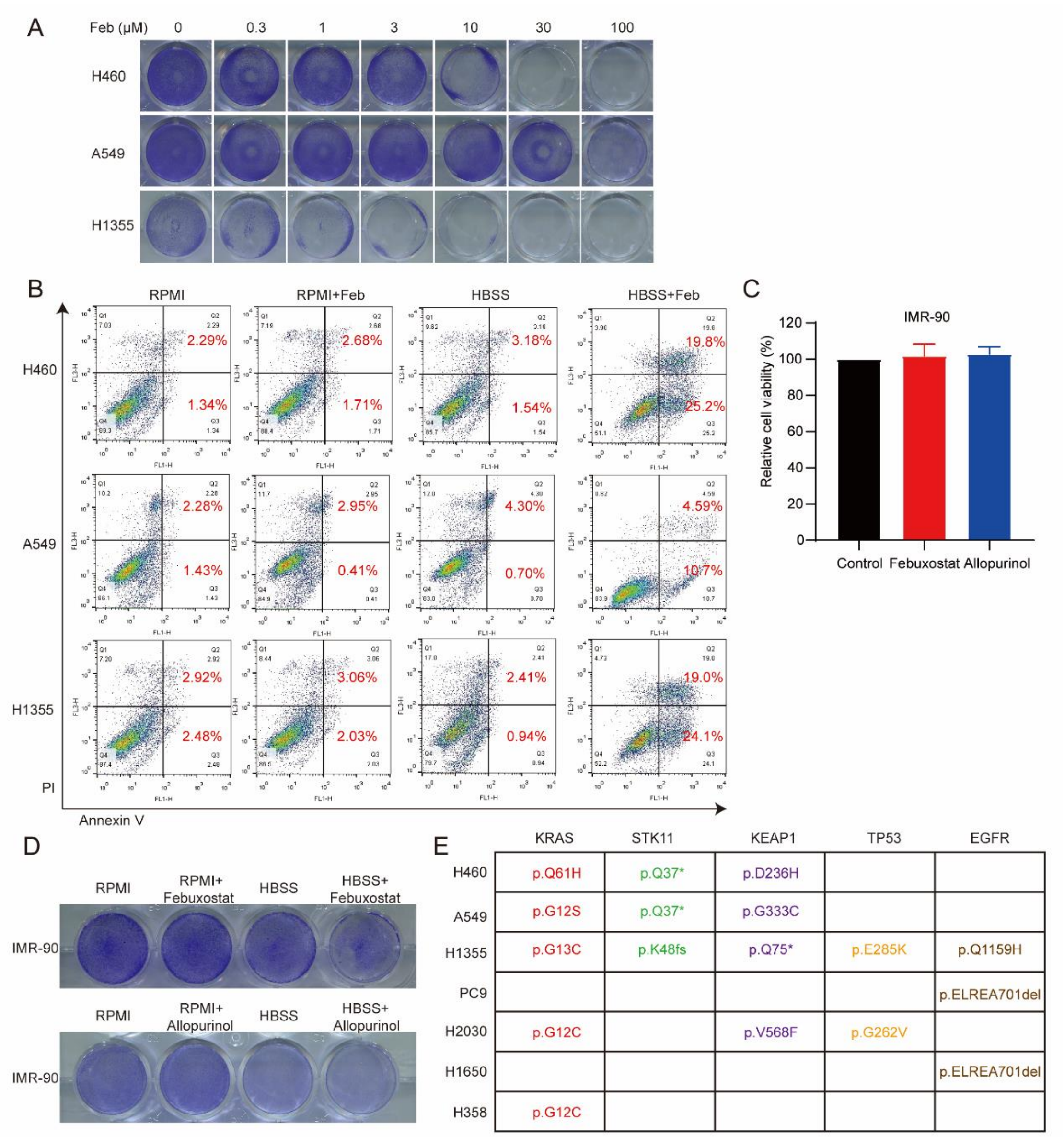

	KRAS	STK11	KEAP1	TP53	EGFR
H460	p.Q61H	p.Q37*	p.D236H		
A549	p.G12S	p.Q37*	p.G333C		
H1355	p.G13C	p.K48fs	p.Q75*	p.E285K	p.Q1159H
PC9					p.ELREA701del
H2030	p.G12C		p.V568F	p.G262V	
H1650					p.ELREA701del
H358	p.G12C				

Supplemental Figure 2. XDH inhibition abrogated the survival of LUAD cells and induced apoptosis upon starvation.

(A) Clonogenic survival assay of LUAD cells in HBSS in the presence of febuxostat at indicated concentrations (n=3). (B) LUAD cells cultured in RPMI or HBSS were treated with febuxostat (100 μM) for 18 h and stained with propidium iodide (PI) and Annexin V. Apoptosis was measured with a flow cytometry. (C) IMR-90 cells cultured in complete medium were treated with febuxostat (100 μM) or allopurinol (100 μM) for 72 h and cell viability was measured with SRB assay. Data are presented as mean ± SD from three independent experiments. (D) Clonogenic survival assay of IMR-90 cells cultured in

complete medium or HBSS containing 100 μM of febuxostat or allopurinol. Representative images from three independent experiments were shown. (E) Boxes indicated mutations in KRAS, STK11, KEAP1, TP53 or EGFR harboring in LUAD cells utilized in the study.

Supplemental Figure 3

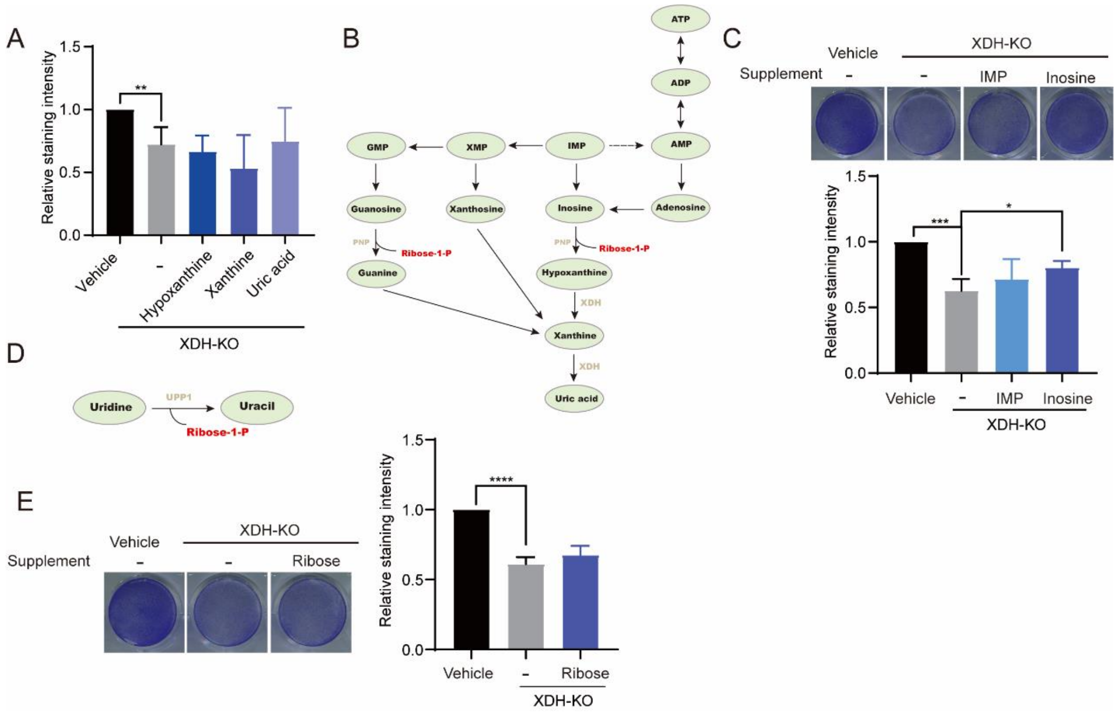

Supplemental Figure 3. Nucleosides rescued the survival of starved LUAD cells upon XDH downregulation.

(A) XDH-knockout H460 cells were cultured in HBSS supplemented with 2 mM of hypoxanthine, xanthine or uric acid. The staining of crystal violet was dissolved in acetic acid and the OD values were measured at 570 nm. **: $p < 0.01$. (B) The diagram of the process of purine degradation. (C) Clonogenic survival assay of XDH-knockout H460 cells cultured in HBSS supplemented with 2 mM of Inosine or IMP. Representative images from three independent experiments were shown. The staining of crystal violet was dissolved in acetic acid and the OD values were measured at 570 nm. *: $p < 0.05$, ***: $p < 0.001$. (D) The diagram of the ribose generated in the uridine degradation. UPP1, Uridine Phosphorylase 1. (E) Clonogenic survival assay of XDH-knockout H460 cells cultured in HBSS supplemented with 2 mM of ribose. Representative images from three independent experiments were shown. The staining of crystal violet was dissolved in acetic acid and the OD values were measured at 570 nm. ****: $p < 0.0001$. Data were presented as mean ± SD.

Supplemental Figure 4

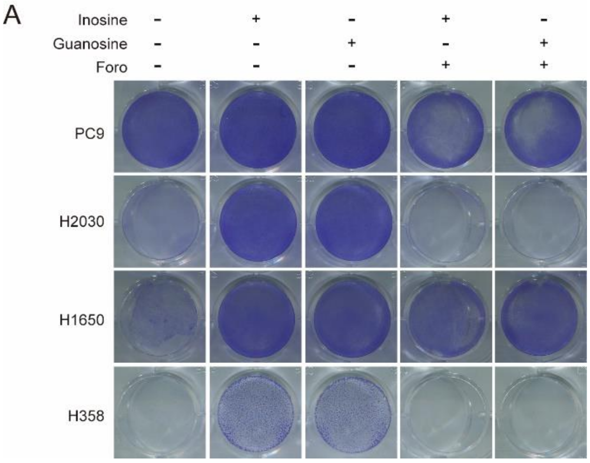

Supplemental Figure 4. Inhibition of purine nucleoside phosphorylase abrogated the survival of starved LUAD cells supplemented with inosine or guanosine.

(A) Clonogenic survival assay of LUAD cells cultured in HBSS or HBSS supplemented with inosine (2 mM), or Guanosine (2 mM) in the presence of forodesine (10 μM) (n=3).

Supplemental Figure 5

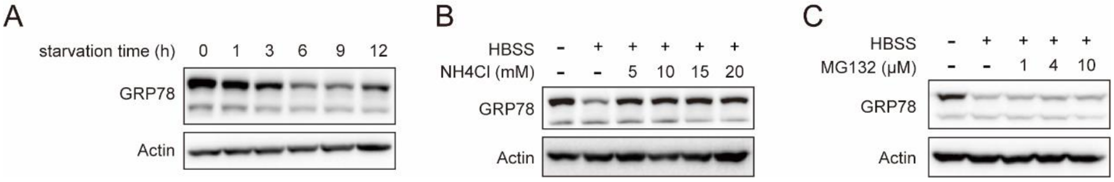

Supplemental Figure 5. Inhibition of XDH suppressed UPR and autophagy in starved LUAD cells.

(A) H460 cells were incubated in HBSS for indicated time and GRP78 was detected by Western blotting. (B-C) H460 cells cultured in HBSS were treated with NH_4Cl (B) or MG132 (C) at indicated concentrations for 9 h. Cell lysates were subjected to Western blotting with indicated antibodies.

Supplemental Figure 6

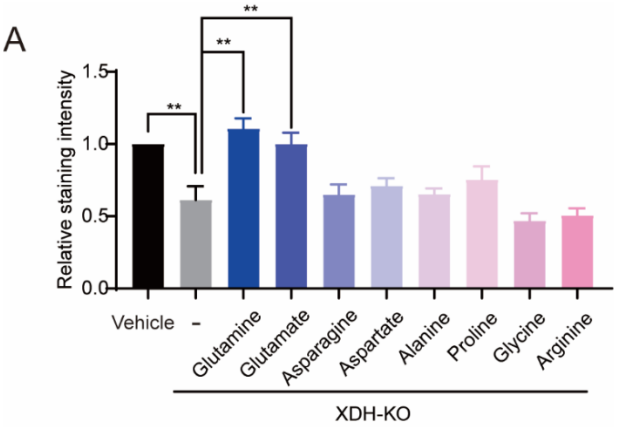

Supplemental Figure 6. Amino acids supplementation rescued the survival of XDH-knockout cells upon starvation.

(A) Clonogenic survival assay of XDH-knockout H460 cells in HBSS supplemented with 2 mM of glutamine, glutamate, asparagine, aspartate, alanine, proline, glycine or arginine. The staining of crystal violet was dissolved in acetic acid and the OD values were measured at 570 nm. **: $p < 0.01$. Data were presented as mean ± SD.

Supplemental Figure 7

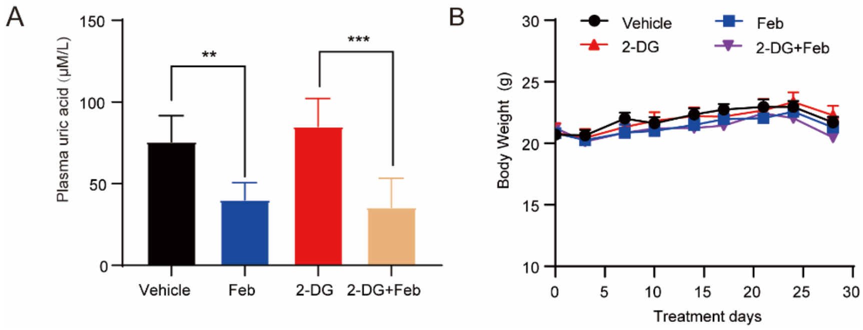

Supplemental Figure 7. The effect of XDH inhibitor combined with 2-DG on the plasma uric acid and body weight of mice.

(A) Uric acid in the blood plasma of mice bearing A549 xenografts treated with febuxostat and 2-DG alone or in combination was measured. Data were presented as mean ± SD. (B) Body weight was measured twice a week after mice bearing A549 xenografts treated with febuxostat (50 mg/kg) and 2-DG (400 mg/kg) alone or in combination. Data were presented as mean ± SEM.
